# Multiscale Investigation on the Performance of Engineered Cementitious Composites Incorporating PE Fiber and Limstone Calcined Clay Cement (LC^3^)

**DOI:** 10.3390/polym14071291

**Published:** 2022-03-23

**Authors:** Guoqiang Gong, Menghuan Guo, Yingwu Zhou, Shuyue Zheng, Biao Hu, Zhongfeng Zhu, Zhenyu Huang

**Affiliations:** 1 College of Civil and Transportation Engineering, Shenzhen University, Shenzhen 518060, China; ggq13501559741@126.com (G.G.); menghuan.guo@szu.edu.cn (M.G.); 1810332085@email.szu.edu.cn (S.Z.); biaohu3-c@szu.edu.cn (B.H.); zhongfeng.zhu@szu.edu.cn (Z.Z.); huangzhenyu@szu.edu.cn (Z.H.); 2 Guangdong Provincial Key Laboratory of Durability for Marine Civil Engineering, Shenzhen University, Shenzhen 518060, China; 3 Key Laboratory for Resilient Infrastructures of Coastal Cities, Shenzhen University, Ministry of Education, Shenzhen 518060, China

**Keywords:** engineered cementitious composites, limestone calcined clay cement, strength, strain, fiber dispersion

## Abstract

Limestone calcined clay cement (LC^3^) is successfully used to fabricate engineered cementitious composites (ECC) exhibiting tensile strength *σ*_tu_ of 9.55 ± 0.59 MPa or tensile strain capacity *ε*_tu_ of 8.53 ± 0.30%. The high tensile strength of the composites is closely related to the improvement of fiber/matrix interfacial bond strength, and the high ductility is attributed to the enhancement of fiber dispersion homogeneity. For the case of ECC incorporating 50% LC^3^, the reduction of initial cracking stress *σ*_tc_ that favors the growth of the crack in a controlled manner also contributes to the improvement of strain hardening behavior. The composition analysis indicates that carboaluminates and additional hydration products including C-(A)-S-H and ettringite are generated, which contributes to the densification of the microstructure of the ECC matrix. The pore structure is thus remarkably refined. Besides, when ordinary Portland cement (OPC) is partly replaced by LC^3^, the consumed energy and equivalent CO_2_ emission decrease, especially the equivalent CO_2_ emission with the reduction ratio attaining 40.31%. It is found that ECC using 35% LC^3^ exhibits the highest mechanical resistance and ECC incorporating 50% LC^3^ shows the highest ductility from the environmental point of view.

## 1. Introduction

Engineered cementitious composites (ECC), a novel class of ultra-high ductile cementitious materials with saturated multiple cracking characteristics, have emerged as a promising option for improving the performance, as well as the sustainability of concrete infrastructures over the past few decades [1]. Designed based on the micromechanics theory, ECC that exhibits obvious strain hardening behavior overcomes the inherent drawback of brittleness in normal concrete [2,3]. Through deliberately tailoring the properties of matrix, fiber, and fiber/matrix interface, ECC, reinforced by a moderate fiber volume of nearly 2%, has an ultra-high tensile strain capacity of more than 3% [4]. ECC possesses the intrinsic crack control capacity, which could efficiently impede the penetration of aggressive ions, thus improving the material/structural durability [5,6,7,8]. The life cycle maintenance and repair needs could be dramatically reduced, and high sustainability is thus achieved [6].

Nevertheless, the fabrication of ECC that consumes a high content of cement not only increases the initial material cost but also compromises the carbon and energy footprints of the composites. To promote the broader application of ECC and enhance the sustainability of ECC-made infrastructure, the usage of environmentally friendly ingredients is highly needed. Substituting cement with supplementary cementitious materials (SCMs) such as ground granulated blast-furnace slag (GGBS) and fly ash (FA) have been considered to be an effective method to mitigate the environmental impacts without compromising the mechanical properties [9,10]. However, the total amount of available fly ash and slag accounts for about 15% of the cement production, and the availability of fly ash keeps decreasing as traditional coal-fired power plants encounter increasingly high environmental pressure [11,12]. Furthermore, the replacement ratio of fly ash as SCM in cement was generally limited to 30%, otherwise, the high dosage of fly ash would have adverse effects on the strength development of ECC [13]. Therefore, it is highly necessary to find alternative cementitious materials.

Limestone calcined clay cement (LC^3^), proposed by Scrivener et al. [14,15], promises to be one of the suitable alternatives. When thermally activated, clay possesses high pozzolanic activities in contact with cement [16,17]. Limestone that acts as an effective filler also participates in the reaction with the reactive components in calcined clay [18,19,20]. The abundant reserves of clayey soils and limestone could ensure the continuous supply [14]. Moreover, the embodied energy and carbon for producing calcined clay and limestone powder are much lower than those for cement production [13]. Several researchers [21,22] have explored the possibility of incorporating LC^3^ in strain-hardening cement-based composites. Liu et al. have explored the compression properties and microstructural characteristics of LC^3^ based-ECC with different polypropylene (PP) fiber content and curing conditions [23]. Yu et al. have used ultrahigh-volume limestone and calcined clay blend to produce ECC, which exhibits tensile strain capacity of 0.57–1.58% and tensile strength of 3.24–5.19 MPa [24]. Ductile LC^3^-ECCs with a tensile strain capacity of 6% are also reported in [25,26]. Although substantial progress has been made, the in-situ fiber distribution state of ECC incorporating LC^3^ has not yet been studied.

Since ECC is designed based on the micromechanical principles, the composite behavior at the macroscale is closely related to the fiber distribution state and the interfacial bond properties between fiber and matrix. Poor fiber dispersion not only has detrimental effects on the mechanical properties of fiber-reinforced cementitious composites but also makes it difficult to establish the link between fiber dispersion and strain hardening properties [27,28]. Thus, a reliable quantification method is highly required to determine the fiber distribution characteristics of ECC [27]. This work aims to investigate the influence of LC^3^ on the fiber distribution homogeneity as well as the fiber/matrix interface and to explore the links between microscale properties and macroscale mechanical performance. Furthermore, LC^3^-ECC designed in this work exhibits not only high tensile strength but also high tensile ductility. A good balance has been achieved between mechanical resistance and deformability.

In this paper, the mechanical performance of ECC incorporating three dosages of LC^3^ (0, 35%, and 50%) was investigated. The cubic compression test and uniaxial tensile test of dog-bone-shaped specimens were performed. The fiber dispersion state was studied by fluorescence technique, and the digital image analysis was carried out. The single fiber pullout test was performed to quantitatively analyze the fiber/matrix interfacial properties. The microstructural characteristics of the ECC matrix were also investigated. Mercury intrusion porosimeter (MIP) test was used to study the influence of LC^3^ on the pore structure of ECC. X-ray diffraction (XRD) and thermogravimetric analysis (TGA) tests were conducted to analyze the phase assemblage of ECC matrix incorporating LC. Through performing the life cycle assessment, the environmental advantages of using LC^3^ in ECC are also discussed.

## 2. Experiment Program

### 2.1. Materials and Mixture Proportion

**Figure 1:**
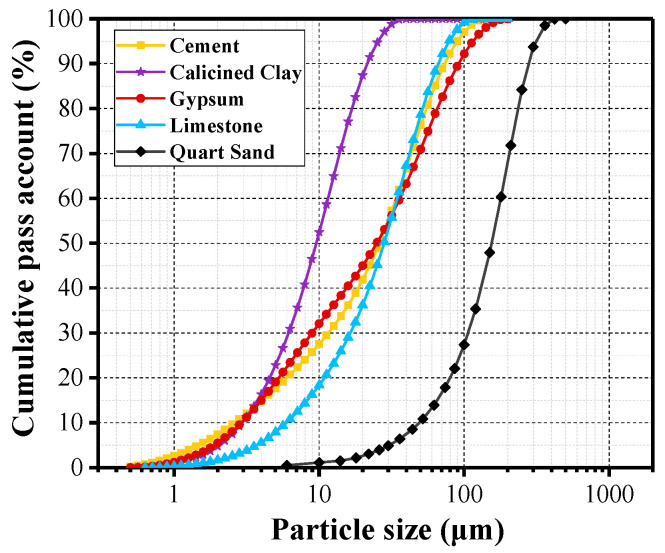
Grading curves of the ingredients.

Reference ECC was composed of ordinary Portland cement (OPC) 52.5R (VCEM P·I), quartz sand, polyethylene (PE) fiber, and water. While for ECC-LC^3^ groups, calcined clay, and limestone powder at the mixing ratio of 2:1 was used to replace OPC [29]. The replacement ratios were, respectively, 35% and 50%, as shown in Table 1. Gypsum was added to adjust the early-age reaction process of aluminates. Polycarboxylate-based high-range water reducer (HRWR), sika viscocrete 3301–40, was added to improve the workability of the fresh mixture. The water to binder ratio was kept being 0.25 for the three studied mixtures. The particle size distribution curves of raw materials, determined by a laser particle analyzer, are demonstrated in Figure 1. Calcined clay was made from kaolin tailings in Maoming, China. Calcined clay mainly contained quartz, illite, and amorphous phases. The chemical composition of all used binders in Table 2 was quantitatively analyzed by the X-ray fluorescence (XRF) technique. ZSX Primus Ⅱ X-Ray Fluorimeter, Rigaku (Tokyo, Japan), was used for XRF testing. PE fiber, purchased from the company QUANTUMETA in Beijing, China, exhibits hydrophobic nature, and its detailed properties are illustrated in Table 3. To ensure the uniform distribution of fibers in the cementitious mixture, the following mixing procedure was employed. Initially, the solid mix, including OPC, calcined clay, limestone powder, gypsum, and quartz sand, was stirred in the mixer at a low speed for 3 min. Afterward, water along with HRWR was poured into the dry blend. The mixture was agitated at a low speed for 5 min and a high speed for 1 min. PE fibers were gradually added to the mixture during the stirring process. Finally, the agitation process lasted for another minute to eliminate the agglomeration of fibers in the slurry. According to GB/T 2419–2005, the fluidity of the fresh mixture was tested. After 24 h, the cast samples were demolded and put in a standard curing room at 23 ± 3 °C and 95% relative humidity until 28 days.

### 2.2. Macroscopic Mechanical Test

**Figure 2:**
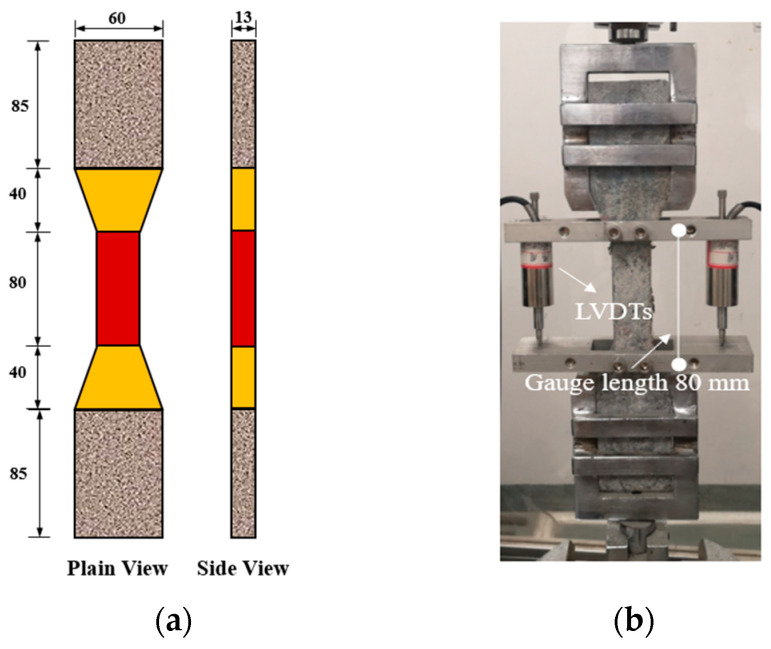
Dog-bone shaped specimen for tensile test: (**a**) geometric size; (**b**) test instrument. (unit: mm).

Three 50 × 50 × 50 mm^3^ cubes were used for compression test according to JGJ/T70−The cubic specimens were loaded at a rate of 0.3 MPa/s by a 2000 kN MTS machine. The average compressive strength of the three specimens was used for further analysis. According to the recommendation of Japan Society of Civil Engineers (JSCE) [30], dog-bone shaped specimens were used to determine the tensile properties of ECC. The geometric size of the specimen is demonstrated in Figure 2a. An MTS Landmark electro-hydraulic servo machine was employed to carry out the uniaxial tensile test. The load was applied at the rate of 1 mm/min. The tensile deformation over the gauge length of 80 mm was measured by two linear variable displacement transducers (LVDTs) installed on the two lateral faces of the specimen, as shown in Figure 2b. For each mixture, at least three specimens were tested. After the test, the tensile cracking patterns of the central narrow part of the specimen were analyzed.

### 2.3. Fiber Distribution Evaluation

**Figure 3:**
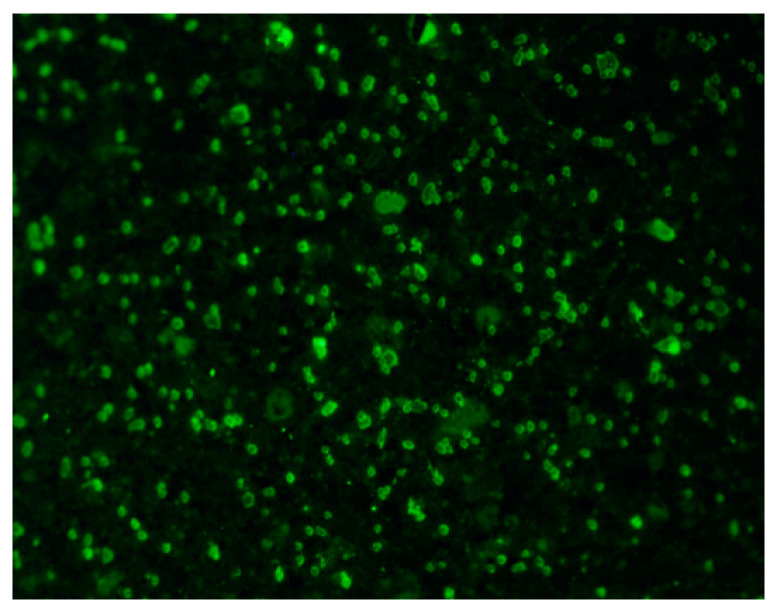
Fluorescence image of the cross-section of the ruptured specimen after tensile test.

Fiber dispersion uniformity plays a vital role in determining the tensile performance of ECC. In this work, the fluorescence technique was used to detect PE fibers in ECC and was employed for quantifying fiber distribution. It is known that non-organic PE fibers could fluoresce and emit green light of 449 nm wavelength when excited by ultra-violet incident light with the wavelength being 385 nm [31]. By separating the emitted fluorescence from the illumination light using a UV filter, fibers appear as greenish dots under the fluorescence microscope while the surrounding matrix appears as dark gray, as illustrated in Figure 3.

After the tensile test, the central loading zone of the specimen was cut into five equal parts, and the cross-section of each part was used for fluorescence analysis. An Olympus BX-51 microscope equipped with an Olympus DP-70 high-resolution digital camera was employed to capture fluorescence images of dog-bone cross-sections. For each cross-section, the whole region was divided into fifteen rectangular zones, and one image was captured for each rectangle by a DP-70 camera [32]. The fiber distribution coefficient α_f_ was then calculated according to the following formula:

(1) $$\alpha_{f}=\exp\left(-\sqrt{\frac{\sum {\left(X_{i}/X_{\mathrm{aversge}}-1\right)}^{2}}{n}}\right)$$

where X_i_ is the number of fibers in the i-th image, determined according to the methodology proposed by Lee et al. [27]; X_average_ is the average number of fibers in all images; and n is the image number. The value of α_f_ tends towards 1 when the fiber dispersion is homogeneous, while the value of α_f_ becomes close to 0 when the fiber dispersion is nonhomogeneous.

### 2.4. Single Fiber Pullout Test

**Figure 4:**
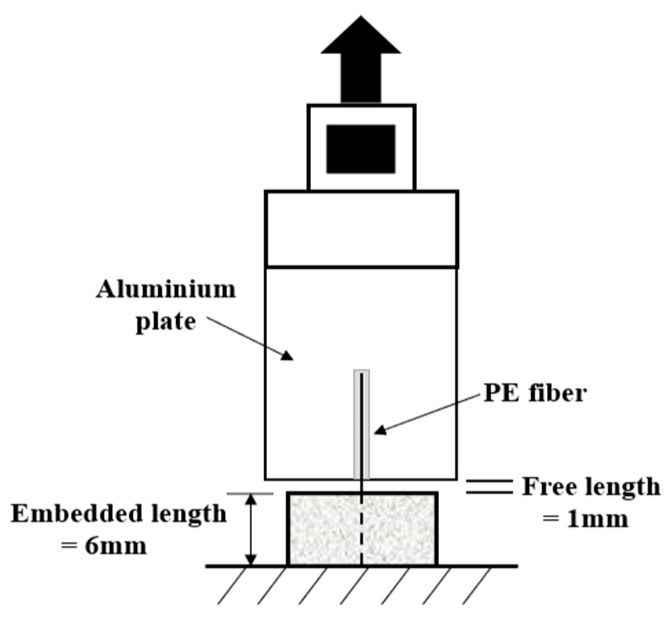
Setup for single fiber pullout test.

To characterize the interfacial properties between PE fiber and LC^3^-based cementitious matrix, the single fiber pullout test was performed. The alignment-controlled PE fiber was taped to a plastic mold and the fresh mixture was poured in. The specimen was demolded after 24 h and then cured in a room environment until 28 days. Through properly cutting in perpendicular to the fiber length direction, the single fiber pullout samples were obtained. The fiber embedment length of each sample was 6 mm, and the fiber-free length was 1 mm. The pullout test was carried out using a universal testing machine. The fiber-free end was glued to an aluminum plate connected to the load cell and the bottom of the single fiber pullout specimen was taped to the base plate of the device. The pullout force was measured by a 5 N load cell. The whole test setup was illustrated in Figure 4. For each mixture type, at least 3 specimens were tested. During the test, the displacement-controlled load was applied at a rate of 1 mm/min.

### 2.5. Microstructure Characterization Test

To characterize the influence of LC^3^ binder system on the micropore structure of ECC matrix, MIP was used to determine the porosity and pore size distribution curves of the matrix. Poromaster GT-60 Mercury Intrusion Porosimeter, Quantachromre (Boynton Beach, FL, USA), was used for MIP testing. The small sample particles were immersed in absolute ethyl alcohol to terminate hydration and then vacuum oven-dried at 60 ℃ for 24 h before test. The hydration phases of the cementitious matrix were investigated by carrying out XRD test. D8 ADVANCE X-Ray Diffractometer, BRUKER, AXS (Karlsruhe, Germany), was used for XRD testing. Samples were vacuum dried and then grounded into fine powders. Cu Kα radiation (λ = 1.54 Å) was performed at 40 kV and 40 mA, and the scan speed was set to be 0.02°/step. The TGA test was also conducted to study the phase assemblage of ECC matrix. STA409PC Simultaneous Thermal Analyzer, Netzsch (Nedgex GmbH, Selb, Germany), was used for TGA testing. The dried sample particles were heated from the range of 30 ℃ to 1000 ℃under a nitrogen atmosphere at a rate of 10 ℃/min. The weight loss was analyzed afterward.

### 2.6. Methodologies of Environmental Impact Assessment

Life cycle assessment (LCA) is conducted to evaluate the potential environmental impact of ECC, for which four steps, including system boundary definition, inventory analysis, assessing impacts, and interpretation of results, are conducted based on the guideline of ISO 14040. This work focused on the environmental impact caused by the raw material processing of ECC. GaBi software (10.5.1.124, Sphera Solutions GmbH, Leinfelden-Echterdingen, Germany), that has a self-developed database was used for LCA analysis. This database could connect with the commonly used international databases, which enhances the correctness of data and ensures the reliability of LCA results.

## 3. Microstructural Analyses

### 3.1. Pore Structure Analysis

**Figure 5:**
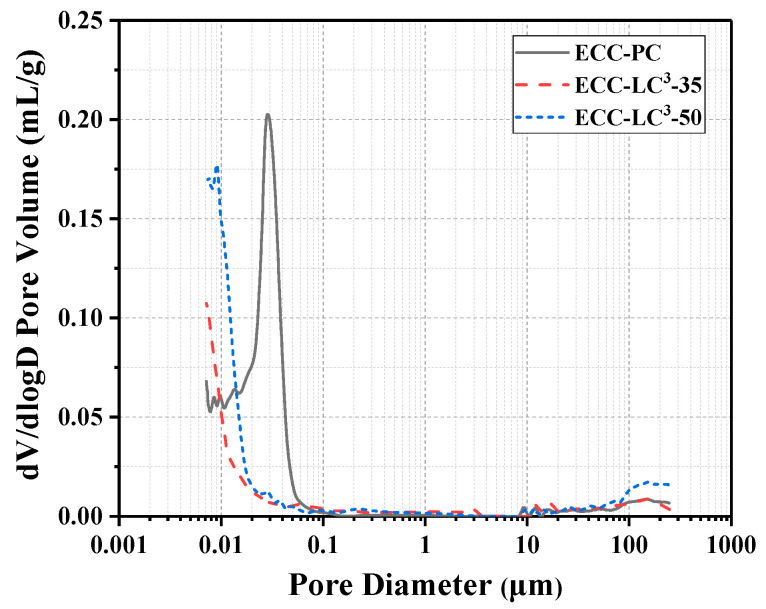
Pore distribution.

**Figure 6:**
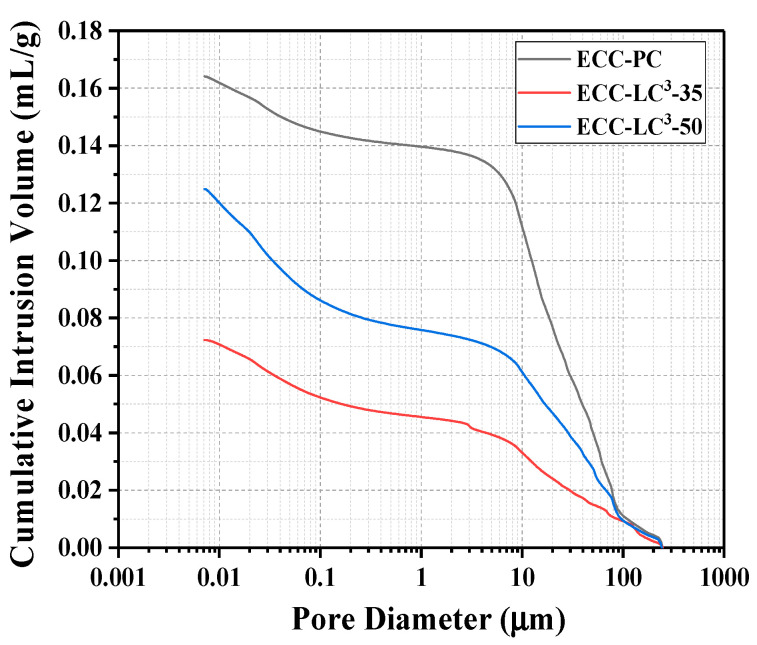
Cumulative pore volume.

The micropore structure of the cementitious matrix is one of the key factors influencing the macroscopic mechanical performance of ECC. In this work, the influence of LC^3^ on the pore structure characteristics of the cementitious binder of ECC was investigated with MIP analysis. Figure 5 demonstrates the pore size distribution of the three kinds of binder systems. It is remarked that the distribution peak shifts towards the smaller pore size regions with the incorporation of LC. The pore refinement of ECC-LC^3^–35 matrix is the most remarkable. Since the pore size distribution of cementitious binder is closely related to the features of hydration products, the reduction of pore size owing to the addition of LC^3^ implies that the hydrate phase assemblage is altered. Figure 6 shows that the total porosity is significantly reduced owing to the incorporation of LC^3^ [33]. Compared to the reference group, the reduction ratios for ECC-LC^3^–35 and ECC-LC^3^–50 reach 55.92% and 24.18%, respectively, as illustrated in Table 4. The porosity of ECC-LC^3^–35 is the lowest while its compressive strength is the highest. The substitution of OPC by 35% LC^3^ demonstrates a more significant influence on the micropore structure of ECC. The phase assemblage of the cementitious composites of ECC incorporating different dosages of LC^3^ will be investigated in the following section.

### 3.2. XRD Results

**Figure 7:**
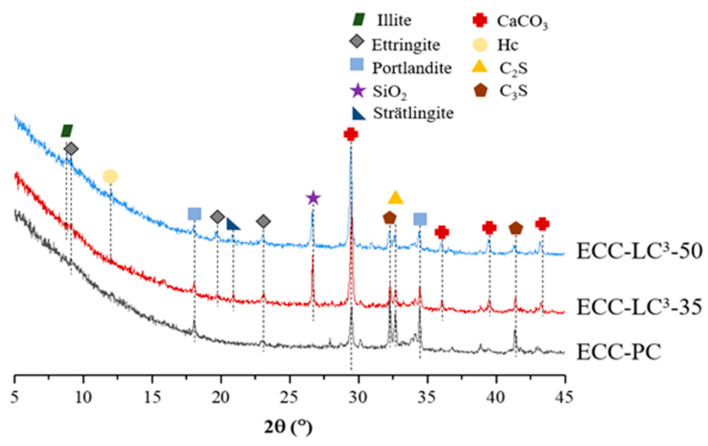
XRD patterns.

The chemical phase compositions of the three kinds of ECC are presented in Figure 7. The phase assemblage of ECC using LC^3^ is different from that of the reference ECC. For LC^3^-based blends, hemi-carboaluminates (Hc) are observed, which are generated due to the reaction between the aluminates, in both calcined clay and cement, and CaCO_3_ from limestone. Since calcined clay is composed of quartz, illite, and amorphous phases, the first two of which are observed in the XRD patterns. For the LC^3^-based paste matrix, the peaks of Ca(OH)_2_ are remarkably reduced with the usage of calcined clay and limestone, which could be attributed to the pozzolanic reaction between Ca(OH)_2_ and the reactive silica and alumina phases in calcined clay. Moreover, the presence of Ca(OH)_2_ is obligatory for the formation of carboaluminates. As limestone introduces a large amount of calcite, the intensity of CaCO_3_ peaks rises with the increase of LC^3^ replacement ratio. In contrast, the peaks corresponding to C_2_S and C_3_S are lowered owing to the dilution effect, i.e., the substitution of OPC by LC^3^ reduces the effective water/cement ratio. The hydration process is accelerated with the usage of calcined clay and limestone, and the hydration degree of cement clinker is thus improved [34]. Besides, as pointed out by Dhandapani and Santhanam [35], calcium aluminum silicate hydrates (C-A-S-H) instead of calcium silicate hydrates (C-S-H) are formed due to the pozzolanic reaction between metakaolin in calcined clay and portlandite. Strätlingite is also observed in LC^3^-based groups. The compositional difference could be the reason for more compact microstructure with refined capillary pore space.

### 3.3. TGA Results

**Figure 8:**
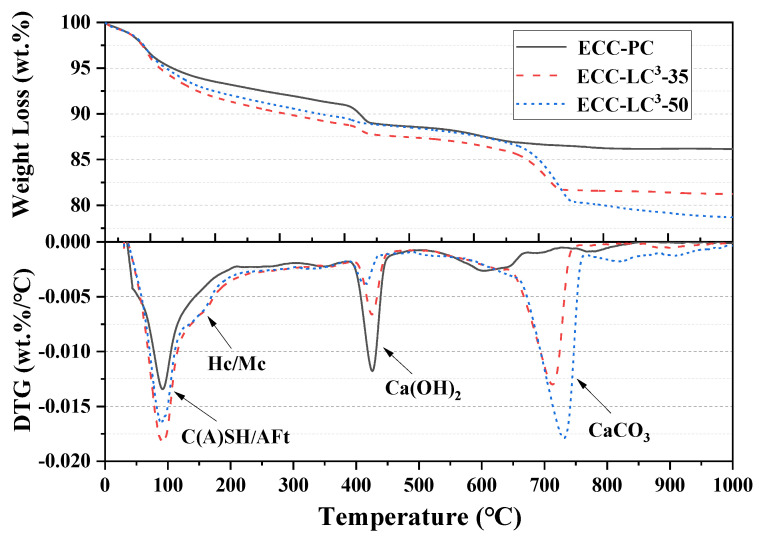
TGA and DTG curves.

Figure 8 presents the TGA and different thermal gravimetric (DTG) curves of the three kinds of specimens. During the heating process, hydrates and minerals undergo various thermal reactions, and these reactions are generally associated with weight changes. The temperatures at which these processes occur are typical for the hydrate or mineral. In the present binder systems, the first peak of mass loss around 100 °C is related to the loss of water from C-(A)-S-H and Aft. The peak intensity of LC^3^-based groups is significantly enhanced compared to the reference OPC, which implies that a large amount of additional hydration products is generated owing to the incorporation of calcined clay and limestone powder. The additional volume of hydrated phases fills the interstices left by the unreacted particles and contributes to the densification of the microstructure of the ECC matrix. For the LC^3^-based matrix, the magnitude of the second peak of mass loss around 170 ℃ is quite modest, which corresponds to the loss of water from carboaluminates. The peak of mass loss around 460 ℃ is related to the dehydroxylation of Ca(OH)To quantify the pozzolanic reactivity of calcined clay, the Ca(OH)_2_ content was determined according to the method proposed by Weerdt et al. [36]. The results were normalized to the cement dosage, and the relative Ca(OH)_2_ content is presented in Table 5. It is remarked that the Ca(OH)_2_ is largely consumed by the pozzolanic reaction and that its content is significantly reduced with the increase of LC^3^ dosage. As for the peaks of mass loss between 700 and 800 °C, they are related to the decarbonation of calcium carbonate and the release of CO. These peaks are significantly enhanced for the LC^3^-based groups owing to the incorporation of limestone powder. To summarize, the TGA results confirm that the phase assemblage of LC^3^ binder system is altered.

## 4. Flowability

The flowability of the three types of ECC is presented in Table 6. As calcined clay exhibits high specific surface area and layered particle structure, the addition of LC^3^ reduces the flowability of ECC and increases the water demand [37]. The superplasticizer dosage was adjusted to maintain similar flowability of the three kinds of ECC.

## 5. Mechanical Properties

### 5.1. Compressive Strength

**Figure 9:**
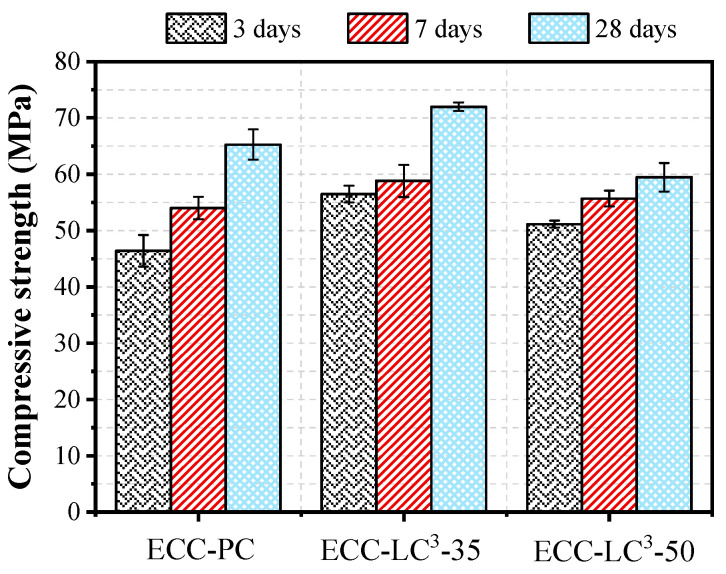
Compressive strength.

The compressive strength of the three studied ECC types is illustrated in Figure 9. At 3 days, the strength increases with the incorporation of LC^3^, and the increasing ratios attain 21.74% and 10.23%, respectively, for ECC-LC^3^–35 and ECC-LC^3^-A similar trend was observed at 7 days. While at 28 days, the compressive strength first increases and then decreases with the dosage of LC. For ECC-LC^3^–35, the increasing ratio is 10.28%, and for ECC-LC^3^–50, the reduction ratio is 8.89%. The strength improvement at an early age could be attributed to the high pozzolanic activity of calcined clay, which exhibits finer particle size, as shown in Figure 1 [38]. The active components in calcined clay react with the hydration product Ca(OH)_2_ to form additional C-A-S-H gels. However, the dilution effect aggravates with the increase of the substitution ratio of OPC by LC. The available Ca(OH)_2_ content diminishes, and the pozzolanic reaction is limited, which explains the reduction of compressive strength of ECC-LC^3^–50 at 28 days. This phenomenon implies that there exists an appropriate replacement ratio of OPC by LC^3^.

### 5.2. Tensile Properties

**Figure 10:**
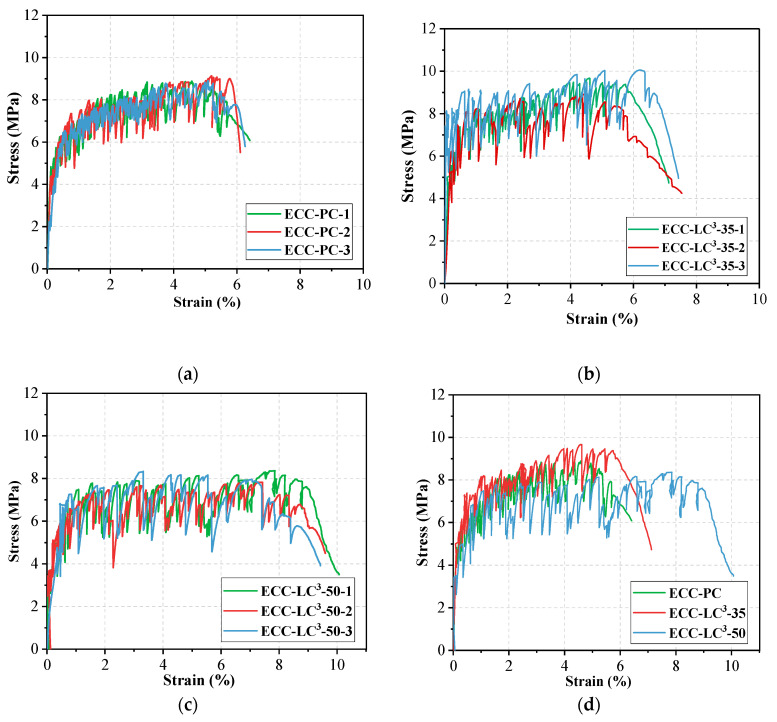
Tensile stress-strain curves of the three types of ECC: (**a**) ECC-PC; (**b**) ECC-LC^3^–35; (**c**) ECC-LC^3^–50; (**d**) representative curve.

**Figure 11:**
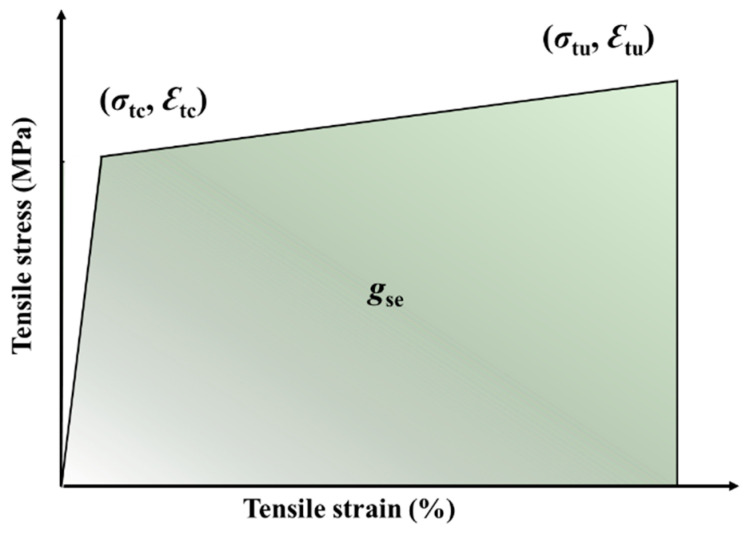
Bilinear model for the tensile stress-strain curve of ECC.

**Figure 12:**
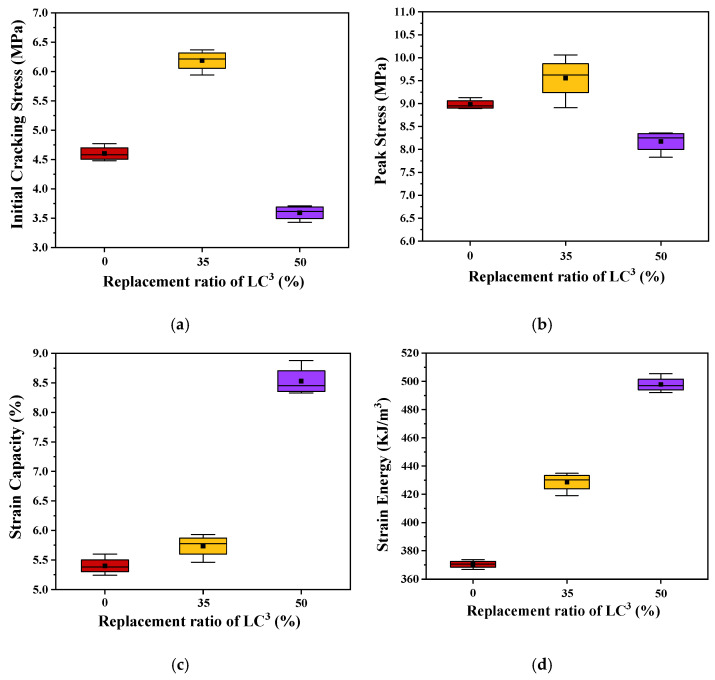
Characteristic parameters of the three types of ECC: (**a**) initial cracking stress; (**b**) peak stress; (**c**) strain capacity; (**d**) strain energy.

The tensile stress-strain curves of the three kinds of ECC are presented in Figure 10. It is noted that LC^3^-based ECCs exhibit strain hardening behavior. The strain capacity of the designed ECC, being about 8%, is hundreds of times higher than that of ordinary concrete and fiber reinforced concrete. The shape of the tensile stress-strain curve of ECC looks more like that of steel than normal concrete. A bilinear model could be used to describe the stress-strain relationship, as shown in Figure 11. During the tensile loading process, the stress shed by the cementitious matrix is gradually transferred to fiber, the slipping and rupturing of fiber lead to the occurrence of stress fluctuation, as shown in Figure 10. The ultimate tensile strength *σ*_tu_ of ECC-LC^3^–35 attains 9.55 ± 0.59 MPa, and the ultimate tensile strain *ε*_tu_ of ECC-LC^3^–50 reaches 8.53 ± 0.30%. The results imply that the combination of calcined clay and limestone could be successfully used to fabricate ECC exhibiting both high strength and high ductility. The initial cracking stress *σ*_tc_, referring to the turning point of the tensile stress-strain curve, and the strain energy *g*_se_, referring to the enclosed area of the ascending branch of the stress-strain curve, are investigated. The trends of the two tensile characteristic parameters are demonstrated in Figure 12.

It is remarked that *σ*_tc_ firstly rises and then declines with the increase of LC^3^ dosage. A similar trend is also observed for *σ*_tu_. As explained in the previous section, the substitution of OPC by LC^3^ reduces the effective water/cement ratio, and the reduction of the content of reactive clinker lowers the build-up of hydrated phases, which is detrimental to the strength development of ECC. Thus, the mechanical resistance of ECC-LC^3^–50 is inferior compared to that of the reference and ECC-LC^3^–35 groups. In contrast, the strain capacity *ε*_tu_, as well as the strain energy *g*_se_ of ECC-LC^3^–50, is the highest. The strain hardening features of ECC are largely enhanced owing to the addition of LC. For ECC-LC^3^–50, the increasing ratio of *ε*_tu_ reaches about 57.96% by comparison with that of the OPC-based ECC. According to the micromechanics-based design theory of ECC, the tensile stress *σ*_cr_ to initiate a crack from a pre-existing flaw must be below the bridging capacity of the fibers *σ*_0_ crossing that crack. Thus, the initiated crack will not result in catastrophic loss of load carrying capacity. A high (*σ*_0_/*σ*_cr_) ratio is preferred in order to meet this requirement. The microcrack density could increase with increasing tensile load. In this study, the significant improvement of the strain capacity could be attributed to the relatively low initial cracking stress *σ*_tc_ which favors the growth of cracks in a controlled manner and benefits the formation of multiple cracking without causing catastrophic brittle failure [39]. While for ECC-LC^3^–35, although the initial cracking stress *σ*_tc_ is higher than that of the reference group, the fibers are more homogeneously distributed in cementitious matrix, as elucidated in the following section. The high uniformity of fiber dispersion benefits the improvement of the strain hardening behavior of ECC.

**Figure 13:**
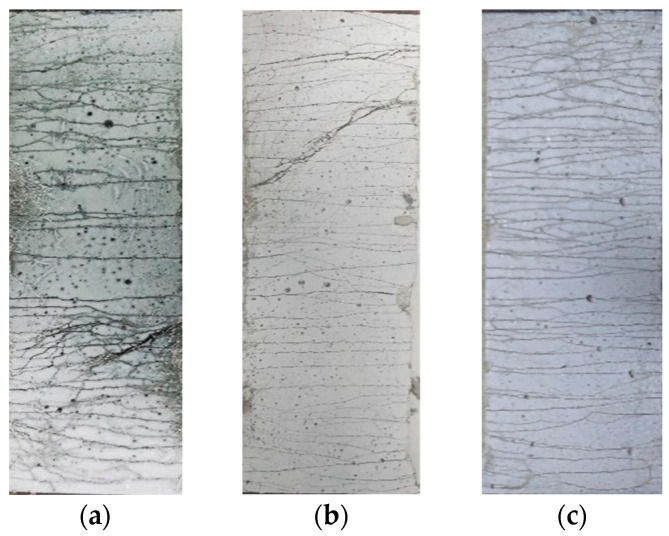
Crack patterns of the three types of ECC after the rupture of the specimen: (**a**) ECC-PC; (**b**) ECC-LC^3^–35; (**c**) ECC-LC^3^–50.

After the tensile test, the crack pattern images of the three types of ECC are captured, as shown in Figure 13. All three types of ECC possess multiple cracking characteristics, and a considerable amount of closely distributed microcracks is generated. For each mixture, the number of cracks (*N*c) in the central gage measured zone is visually counted, as shown in Table 7. It is remarked that *N*c increases with the increment of the substitution ratio of OPC by LC^3^, which corresponds well with the tendency of strain capacity. The average crack space (*S*c), defined as *S*c = 80/*N*c, is calculated. The results illustrate that Sc drops with the replacement ratio of LC. The average crack width (*W*c) is defined as the ratio of the elongation to the number of cracks, and the value of *W*c is found to rise first and then reduce with the increasing of LC^3^ dosage. ECC-LC^3^–35 group exhibits excellent crack control capacity. The tight cracks can effectively enhance the resistance of ECC to chloride diffusion and thus improve the durability of the composites [40,41].

### 5.3. Fiber Distribution Analyses

The fiber dispersion coefficients are summarized in Table 8. Unexpectedly, the fiber dispersion homogeneity is found to rise with the substitution of OPC by LC^3^ through the increase ratio does not show a linear relationship with the replacement ratio of OPC by LCAs pointed out by Cao et al. [42], the rheology of ECC mixture can affect fiber dispersion, the fluorescence analyses carried out in this work imply that the rheological performance of LC^3^-based ECC is adequately ameliorated. When extra HRDR is added to the mixture of LC^3^-based ECC, the active components in calcined clay could act as thixotropic additives [43,44], which modifies the viscosity of the cementitious slurry. The results of fluorescence analysis indicate that the combined action of calcined clay and HRDR positively alters the flocculated structure formed by cement paste and that the flowability, as well as the rheology properties of the composites are improved. Correspondingly, the fiber dispersion uniformity is enhanced, which in turn contributes to the improvement of the strain capacity of LC^3^-based ECC, as illustrated in the previous section. The study of Wu et al. [45] also demonstrated that the tensile ductility of ECC increases with the increase of fiber distribution coefficient, which coincides with the findings in this work. In brief, the macroscopic tensile performance of LC^3^-ECC is closely related to the fiber/matrix interfacial bond properties as well as the fiber distribution homogeneity.

### 5.4. Fiber/Matrix Interfacial Properties

**Figure 14:**
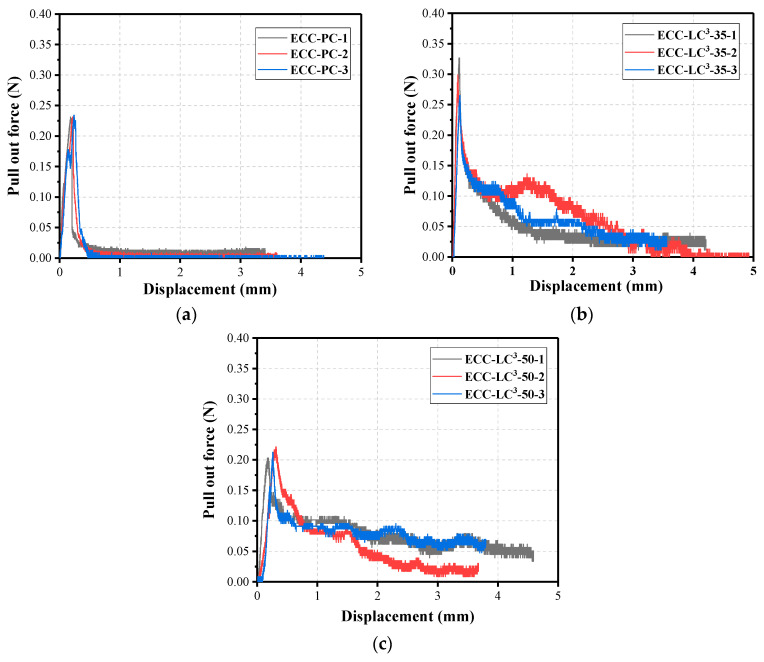
Single fiber pullout curves: (**a**) ECC-PC; (**b**) ECC-LC^3^–35; (**c**) ECC-LC^3^–50.

For the three studied ECC matrix types, the force-displacement curves of the single fiber pullout test are depicted in Figure 14. The slight slip-hardening behavior was observed after the debonding between fiber and matrix. The single fibers were pulled out from the embedded matrix. Considering the hydrophobic nature of PE fiber, the chemical bonding between fiber and matrix could be neglected. The applied external fiber load is dominantly resisted by the interfacial frictional stress between fiber and matrix. The frictional bond strength *τ*_0_ was calculated according to the following equation [46]:

(2) $$\tau_{0}=\frac{P_{\max}}{\pi d_{f}L_{f}}$$

where *P*_max_ is the peak value of fiber pullout force; *d*_f_ is the diameter of fiber; *L*_f_ represents the embedded length of fiber.

Table 9 depicts the obtained results. It is remarked that the frictional bond strength *τ*_0_ demonstrated firstly an increasing and then a decreasing tendency with the incorporation of LC^3^, which is consistent with the trend of the tensile strength at the composite scale. The interfacial stress between PE fiber and LC^3^–35-ECC matrix, being the highest, contributes to the achievement of the highest tensile strength at the macroscopic scale. The interfacial properties are directly related to the behavior of ECC at the composite scale. The underlying mechanism for the superior mechanical performance of LC^3^–35-ECC will be discussed in the following section.

### 5.5. Environmental Impact Assessment

**Figure 15:**
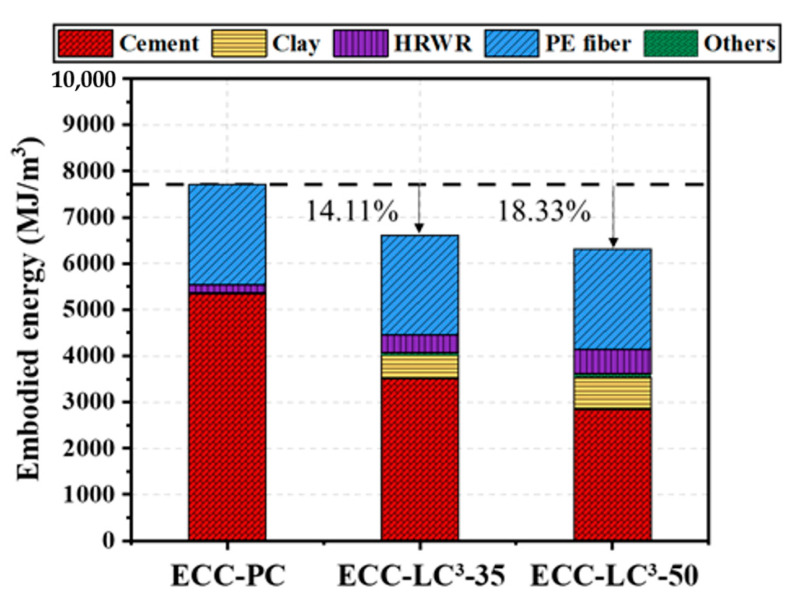
Summarization of the consumed energy by each component of ECC.

**Figure 16:**
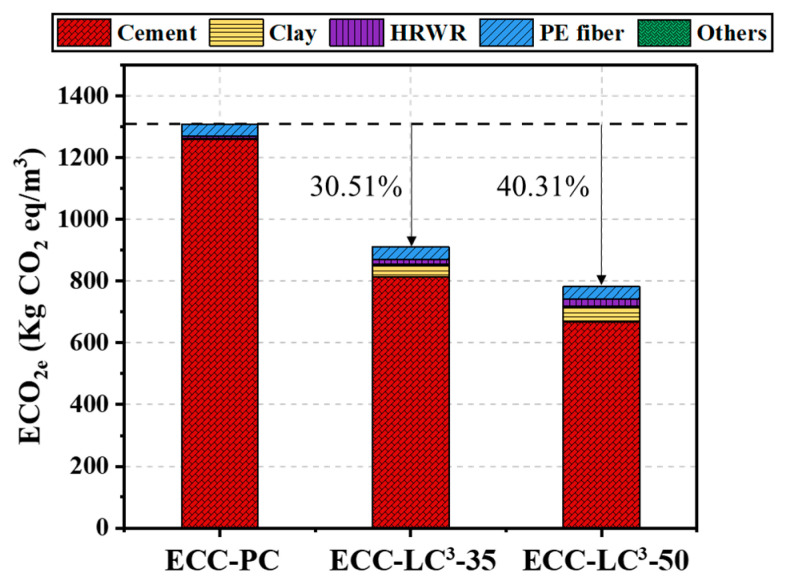
Summary of the GWP by each component of ECC.

**Figure 17:**
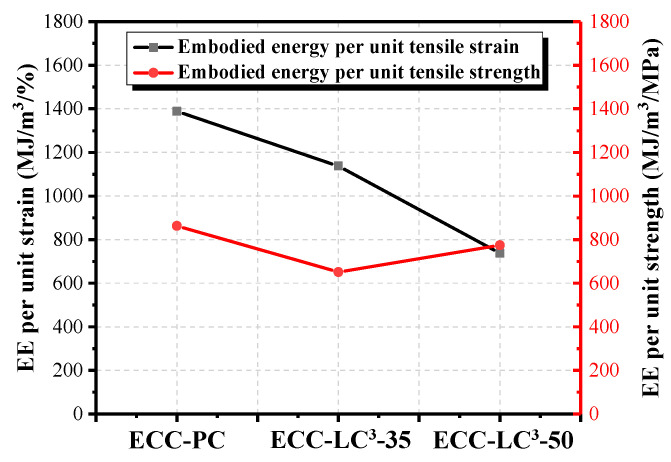
Comparison of the energy consumption of ECC per unit tensile strain and per unit tensile strength.

**Figure 18:**
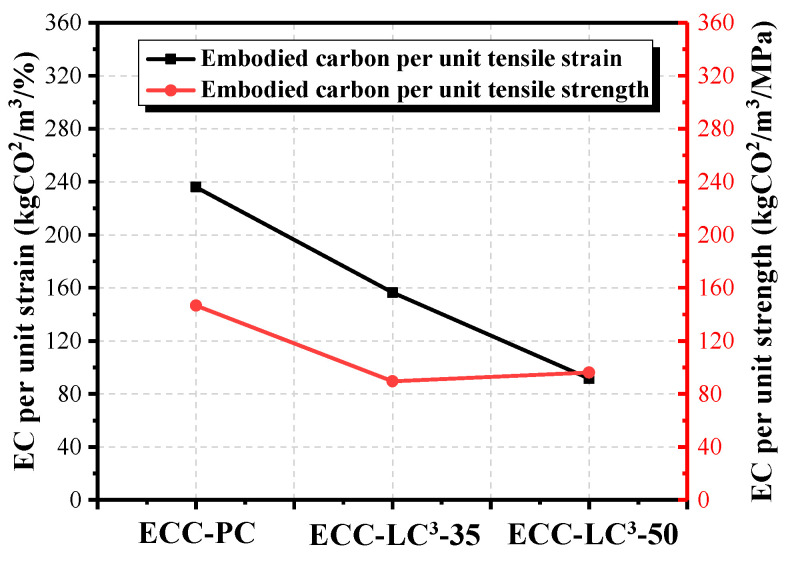
Comparison of the equivalent CO_2_ emission of ECC per unit tensile strain and per unit tensile strength.

The life cycle inventories of cement, calcined clay, gypsum, limestone, water, quartz sand, and HRWR were adopted from the database of GaBi software (10.5.1.124, Sphera Solutions GmbH, Leinfelden-Echterdingen, Germany). As the life cycle inventory of PE fiber is not available in Gabi, the data reported in the literature [47] were used to establish an equivalent inventory. Two environmental impact criteria, i.e., energy and global warming potential (GWP), were calculated by using the Environment Footprint 2.0 method in GaBi software. The obtained results are presented in Figure 15 and Figure 16. It is noted that the environmental impact of cement among all the components of ECC is the highest. The contribution of cement to energy consumption accounts for 69.45% while it reaches even 96.34% to equivalent CO_2_ emission in OPC-based mixture. PE fiber ranks only second to cement although its dosage is quite low. When OPC is partly replaced by LC^3^, the two studied environmental impact criteria descend, especially the GWP with the reduction ratio attaining 40.31%. However, since the incorporation of LC^3^ requires a higher dosage of HRDR, the environmental impact of the ECC mixture caused by HRDR also rises with the substitution ratio of OPC by LC. The contribution of HRDR to energy consumption is more remarkable. Furthermore, both the energy consumption and the equivalent CO_2_ emission per unit strain and unit strength were calculated, as demonstrated in Figure 17 and Figure 18. The energy consumption per unit strain and the equivalent CO_2_ emission per unit strain reduces almost linearly with the increasing of LC^3^ dosage, while the energy consumption per unit strength and the equivalent CO_2_ emission per unit strength show first a descending and then a slight rising trend. It could be concluded that ECC using 35% LC^3^ exhibits the highest mechanical resistance and ECC incorporating 50% LC^3^ shows the best deformability from the environmental point of view.

## 6. Conclusions

A comprehensive investigation concerning the macroscopic mechanical properties, matrix/fiber interfacial properties, microstructural characteristics, fiber distribution homogeneity, and the potential environmental impact of ECC incorporating different dosages of LC^3^ is carried out. The following conclusions can be drawn.

(1) The pozzolanic reactivity of calcined clay plays a dominant role in determining the compressive strength at an early age, while, at 28 days, the compressive strength shows first a rising and then a descending trend with the increasing of LC^3^ replacement ratio. A similar tendency is observed for the tensile strength of the composites as well as the fiber/matrix interfacial bond strength at 28 days. The strength loss of ECC-LC^3^–50 could be attributed to the lowering of available Ca(OH)_2_ content, which limits the pozzolanic reaction.

(2) The strain capacity of ECC is largely enhanced with the incorporation of LC. The increment ratio rises with the increase of LC^3^ dosage, with the strain capacity of ECC-LC^3^–50 being 57.96% higher than that of the OPC-based ECC. The improvement of strain hardening behavior is closely related to the higher fiber dispersion uniformity of LC^3^-based groups. For ECC-LC^3^–50, the relatively low initial cracking stress further favors the growth of cracks in a controlled manner.

(3) The addition of LC^3^ leads to the pore refinement of the ECC matrix, especially for the ECC-LC^3^–35 group, which exhibits the highest strength at 28 days. The composition analysis indicates that carboaluminates are generated owing to the reaction between aluminates and calcium carbonate from limestone. Additional hydration products including C-(A)-S-H and Aft are also formed, which contributes to the densification of the microstructure of the ECC matrix. Besides, the Ca(OH)_2_ content is dramatically reduced while the CaCO_3_ content is remarkably increased.

(4) The results of the life cycle assessment imply that the environmental impact of cement among all the components of ECC is the highest. When OPC is partly replaced by LC^3^, the consumed energy and equivalent CO_2_ emission decrease, especially the equivalent CO_2_ emission with the reduction ratio attaining 40.31%. Besides, the environmental impact of PE fiber and HRDR is non-negligible. By calculating the energy consumption and the equivalent CO_2_ emission per unit strain and unit strength, it is found that ECC-LC^3^–35 exhibits the highest mechanical resistance, and ECC-LC^3^–50 shows the best deformability from the environmental point of view.

## Figures and Tables

**Table 1:** Mixture proportion of ECC (kg/m^3^).

Mixture Type	Cement	Calcined Clay	Gypsum	Water	Limestone	Quatrz Sand	PE	HRWR
ECC-PC	1382	0	0	345	0	500	20	6
ECC-LC^3^–35	905	304	21	345	152	500	20	13
ECC-LC^3^–50	732	415	28	345	207	500	20	18

**Table 2:** Chemical composition of cementitious binders *.

Property	Cement	Calcined Clay	Gypsum	Limestone
MgO (%)	1.775	0.307	3.394	0.769
Na_2_O (%)	0.281	-	-	-
Al_2_O_3_ (%)	3.509	39.405	5.828	0.130
SiO_2_ (%)	15.406	53.732	14.046	0.309
P_2_O_5_ (%)	0.065	0.037	0.050	-
SO_3_ (%)	4.212	0.087	35.867	-
K_2_O (%)	0.0950	4.229	1.403	0.040
CaO (%)	69.862	0.102	37.048	98.715
Fe_2_O_3_ (%)	3.741	2.056	2.032	-
CuO (%)	0.029	-	-	-
ZnO (%)	0.100	-	-	-
SrO (%)	0.070	-	0.333	0.037
Rb_2_O (%)	-	0.037	-	-
Y_2_O_3_ (%)	-	0.001	-	-
ZrO_2_ (%)	-	0.008	-	-

* XRF analysis carried out by ZSX Primus II X-Ray Fluorimeter, Rigaku.

**Table 3:** Properties of PE fibers *.

PE fiber	
Length *L*_f_, mm	18
Diameter *d*_f_, μm	25
Aspect ratio *L*_f_ /*d*_f_	720
Fiber strength, MPa	2900
Modulus of elasticity, GPa	116
Specific gravity, g/cm^3^	0.97
Melting temperature, °C	150

* Provided by the supplier of PE fiber, the company QUANTUMETA in Beijing, China.

**Table 4:** Porosity of ECC matrix.

Mixtures ID	Porosity (%)
ECC-PC	16.38
ECC-LC^3^–35	7.22
ECC-LC^3^–50	12.42

**Table 5:** Relative content of Ca(OH)_2_ calculated based on DTG curves.

Mixtures ID	CH Content (%)
ECC-PC	10.15
ECC-LC^3^–35	5.67
ECC-LC^3^–50	2.41

**Table 6:** Flowability.

Mixture ID	Average Value (mm)
ECC-PC	159
ECC-LC^3^–35	156
ECC-LC^3^–50	151

**Table 7:** Cracking characteristics.

Mixtures ID	*N*_c_	*S*_c_ (mm)	*W*_c_ (μm)
ECC-PC	43 ± 3	1.87 ± 0.13	125.58
ECC-LC^3^–35	48 ± 5	1.69 ± 0.18	121.04
ECC-LC^3^–50	68 ± 6	1.19 ± 0.10	125.44

**Table 8:** Fiber dispersion coefficient.

Mixtures ID	α_f_
ECC-PC	0.643
ECC-LC^3^–35	0.801
ECC-LC^3^–50	0.781

**Table 9:** Frictional bond strength *τ*_0_. (Unit: MPa).

Mixtures ID	Average Value	Standard Deviation
ECC-PC	0.498	0.023
ECC-LC^3^–35	0.630	0.066
ECC-LC^3^–50	0.434	0.036

## Data Availability

The data presented in this study are available on request from the corresponding author.
